# Non-Canonical Roles for Yorkie and Drosophila Inhibitor of Apoptosis 1 in Epithelial Tube Size Control

**DOI:** 10.1371/journal.pone.0101609

**Published:** 2014-07-18

**Authors:** Renée M. Robbins, Samantha C. Gbur, Greg J. Beitel

**Affiliations:** Department of Molecular Biosciences and Robert H. Lurie Comprehensive Cancer Center, Northwestern University, Evanston, Illinois, United States of America; Cardiff University, United Kingdom

## Abstract

Precise control of epithelial tube size is critical for organ function, yet the molecular mechanisms remain poorly understood. Here, we examine the roles of cell growth and a highly conserved organ growth regulatory pathway in controlling the dimensions of the Drosophila tracheal (airway) system, a well-characterized system for investigating epithelial tube morphogenesis. We find that tracheal tube-size is regulated in unexpected ways by the transcription factor Yorkie (Yki, homolog of mammalian YAP and TAZ) and the Salvador/Warts/Hippo (SWH) kinase pathway. Yki activity typically promotes cell division, inhibits apoptosis, and can promote cell growth. However, reducing Yki activity in developing embryos increases rather than decreases the length of the major tracheal tubes, the dorsal trunks (DTs). Similarly, reduction of Hippo pathway activity, which antagonizes Yki, shortens tracheal DTs. *yki* mutations do not alter DT cell volume or cell number, indicating that Yki and the Hippo pathway regulate cell shape and apical surface area, but not volume. Yki does not appear to act through known tracheal pathways of apical extracellular matrix, septate junctions (SJs), basolateral or tubular polarity. Instead, the Hippo pathway and Yki appear to act downstream or in parallel to SJs because a double mutant combination of an upstream Hippo pathway activator, *kibra,* and the SJ component *sinu* have the short tracheal phenotype of a *kibra* mutant. We demonstrate that the critical target of Yki in tube size control is *Drosophila* Inhibitor of Apoptosis 1 (DIAP1), which in turn antagonizes the Drosophila effector caspase, Ice. Strikingly, there is no change in tracheal cell number in DIAP1 or *Ice* mutants, thus epithelial tube size regulation defines new non-apoptotic roles for Yki, DIAP1 and Ice.

## Introduction

Organs comprised of epithelial tubes that transport gases or fluids are essential to life for most multicellular animals. The function of organs, such as the kidneys, lungs and the vascular system is highly dependent on tubes developing to the proper size [1]. Defective tube size control leads to human diseases such as polycystic kidney disease (PKD), in which tubules enlarge to become cysts that severely impair kidney function [2], [3]. The *Drosophila* tracheal system is an excellent model for studying the complex processes underlying epithelial tube morphogenesis (reviewed in [1], [4]–[7]). The *Drosophila* tracheal system serves as a combined pulmonary and vascular system that directly delivers oxygen to tissues through a ramifying network of epithelial tubes. The tracheal system arises from clusters of cells on the surface of the embryo, and these clusters invaginate, undergo one round of cell division and do not divide again. During invagination, tracheal cells retain their apical surfaces and organize into lumen-containing branches. During later embryonic development (stages 15–17), the large dorsal trunk (DT) tubes elongate by changing cell shape and rearranging cell-cell junctions without increasing cell number [8], [9]. Programmed cell death (apoptosis) plays little, if any, role in tracheal morphogenesis as only 1 cell of the ∼80 cells in a tracheal metamere (segment) undergoes apoptosis in about half the developing metameres [10].

Several pathways have been identified that regulate tracheal tube size [4], [11]–[13]. For reasons that are not clear, SJs feature in several of these pathways. Insect SJs are claudin-containing cell-cell junctions that have the paracellular barrier function of the vertebrate tight junctions [14], [15], but have a basolateral localization and contain conserved basolateral polarity proteins such as Scribbled (Scrib), Discs Large (Dlg), and Yurt (Yrt) [16], [17]. SJs have at least two distinct functions in tracheal tube-size control, neither of which involves the paracellular barrier function [12], [18]. First, SJs are required for the specialized apical secretion of Vermiform (Verm) and Serpentine (Serp), putative chitin deacetylases that are part of a transient, chitin-based, apical extracellular matrix (aECM) whose organization is required to restrict the length of the trachea [19]–[24]. Apical secretion of Verm and Serp is lost in SJ mutants such as *sinuous* (*sinu*), *coracle* (*cora*), and *nervana2* (*nrv2*), which leads to a disorganized ECM and elongated trachea. Second, the basolateral polarity proteins that localize to the SJs antagonize the apical polarity protein Crumbs (Crb), a transmembrane protein that promotes expansion of the tracheal cell apical surface and tube elongation [12], [16].

In addition to the aECM and polarity pathways, it was recently shown that the highly conserved non-receptor tyrosine kinase Src42 is required for the normal surface area growth of the tracheal apical membrane, and to orient apical growth along the length rather than the circumference of the tube [25], [26]. Excessive Src42 activity increases apical surface area in the direction of the length of the tube, which makes tracheal cells more rectangular and increases tracheal length. In contrast, insufficient Src42 activity reduces total apical surface area and remaining membrane growth is misoriented around the circumference of the tube. Thus, Src42 mutant trachea have tracheal tubes that are too short but that are also abnormally large in diameter.

The effectors of aECM, polarity, and Src42 pathways have not yet been determined. However, one possibility is the evolutionarily conserved Hippo/MST pathway, also known as the Salvador/Warts/Hippo (SWH) pathway in *Drosophila* [27]–[29], and its downstream effector, the transcription factor Yki (mammalian homologs YAP and TAZ). These genes play central roles in organ size control in many organisms [28], [30]–[34] and recent findings have linked both Crb and Src to Hippo signaling [35]–[39]. However, the roles the SWH/Hippo pathway and Yki/YAP/Taz in epithelial tubule morphogenesis have not yet been investigated in detail. The core SWH/Hippo pathway is a serine/threonine kinase cascade in which Hippo and its mammalian homologs MST1/2 phosphorylate and activate the Warts/LATS kinases. Full activity of Hippo and Warts requires the scaffolding proteins Sav/Sav1 and Mats/Mob1. Yki/YAP/TAZ are negatively regulated by the Hippo pathway through Warts/LATS phosphorylation of Yki/YAP/TAZ that causes Yki/YAP/TAZ to be localized to the cytoplasm. The core Hippo pathway is regulated by numerous converging inputs that respond to cell-cell interactions, receptor signaling, polarity proteins such as Crb, and apical actin cytoskeletal-associated proteins such as Kibra (Kib) [29], [40], [41].

A conserved function of the Hippo pathway and Yki is to regulate cell death via transcriptional regulation of inhibitors of apoptosis. In flies, Yki activates transcription of Drosophila Inhibitor of Apoptosis (DIAP1), which encodes a protein containing two baculovirus IAP repeats (BIR) domains that bind caspases and pro-apoptotic factors, and a RING domain that mediates ubiquitination of DIAP1 itself as well as caspases and cell death regulators that bind the BIR domains of DIAP1. Expression of DIAP1 inhibits cell death by inactivating the caspases Dronc and Ice. Binding of cell death initiators such as Grim, Reaper or Hid to DIAP1 releases the inhibition of Dronc and Ice, which become proteolytically active. Most commonly, activation of caspases leads to cell death, however there are multiple examples of non-apoptotic functions of caspases in Drosophila and mammals [42]–[45].

In this study, we find that Yki has an unexpected role in controlling cell shape during tracheal morphogenesis. While loss of Yki activity typically decreases organ size, loss of Yki increases tracheal dorsal trunk length without increasing tracheal cell volume or number. Despite this unexpectedly opposite result, Yki nonetheless regulates tracheal tube-size through its well-established downstream mediators *thread*/DIAP1 and the effector caspase Ice. However, loss of Yki, DIAP or Ice does not alter apoptosis in the tracheal dorsal trunk. Thus, Yki, DIAP and Ice act in their typical “cassette”, but together they act in a non-canonical pathway that controls tracheal cell shape and organ morphogenesis independently of apoptosis.

## Results

### Yorkie negatively regulates embryonic tracheal tube length

**Figure 1:**
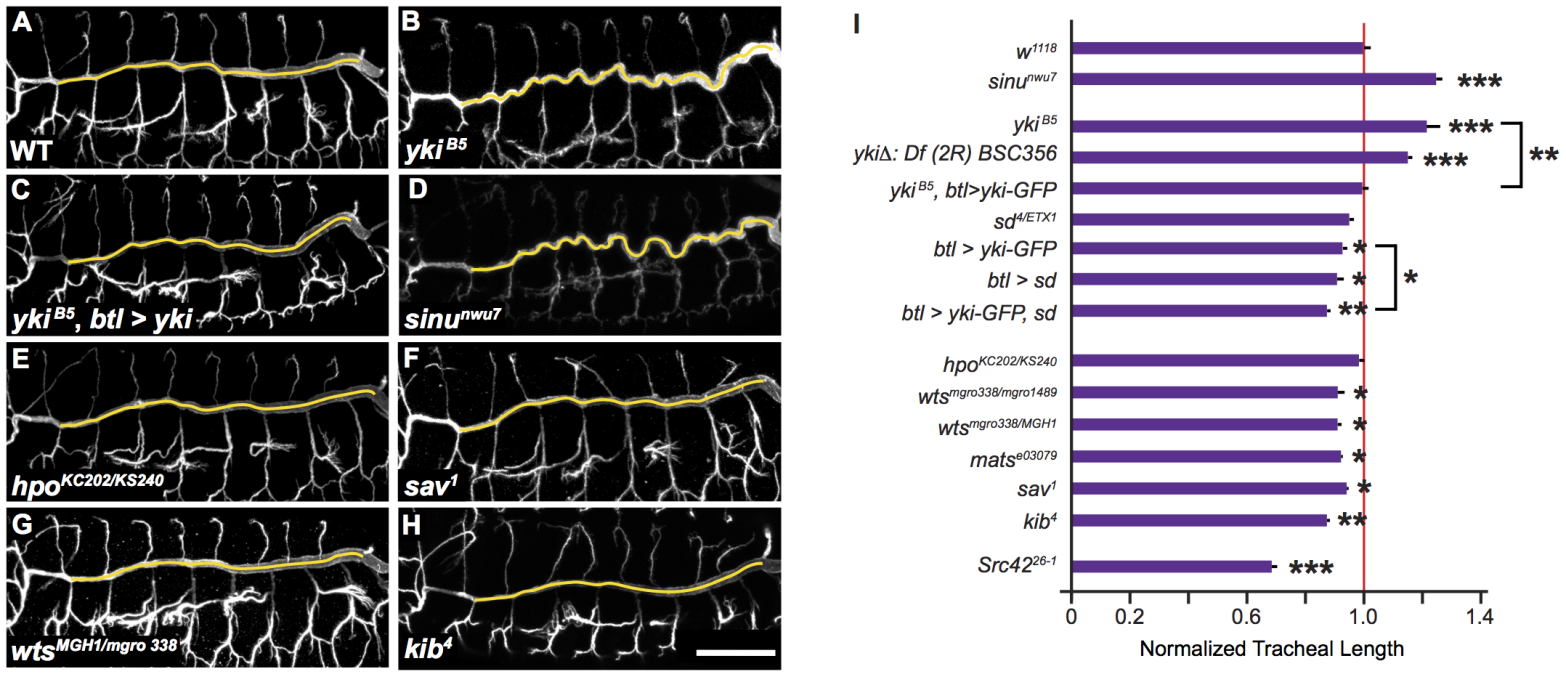
Yorkie and the Hippo Pathway Regulate Tracheal Tube Size. (A–D) Maximum projections of confocal sections showing tracheal lengths. Compared to stage 16 WT embryos (A), *yki^B5^* mutant trachea (B) have an over-elongated phenotype similar to the SJ mutant *sinu* (D). Expression of UAS-*yki* in the trachea of *yki^B5^* mutants rescues tracheal length defects (C). Yellow lines indicate dorsal trunk measurements. Tracheal lumens visualized by anti-2A12 marker staining. (E–H) Although embryos trans-heterozygous for *hpo* mutations have WT tracheal length (E), mutations in Hippo pathway components *sav*, *wts* and *kib* shorten tracheal length (F–H, respectively). (I) Quantification of tracheal length shows that decreased Yki activity lengthens trachea, while increased Yki activity or decreased Hpo pathway activity reduces tracheal length. Tracheal length was measured in three dimensions in confocal stacks, normalized to embryo length and then normalized to WT (*w^1118^*, red line). Error bars show normalized s.e.m.; *: p<0.05; **: p<0.005; ***: p<0.0005. p*-*values determined using Student's *t*-test. Scale bar for (A–G) in (H), 50 µm.

**Figure 2:**
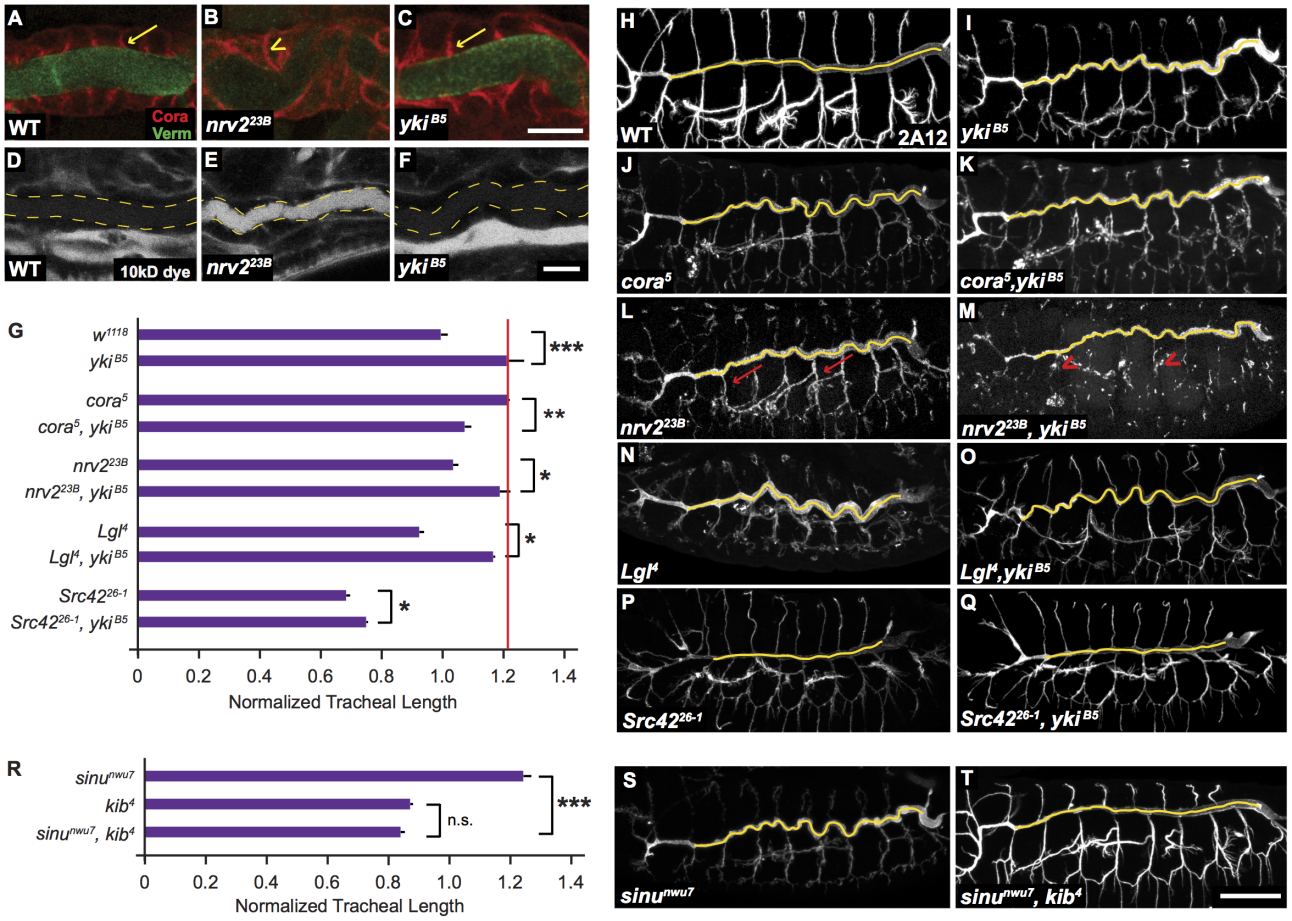
Yki Acts Separately from the Verm/aECM, SJ, Basolateral Polarity, and Src Pathways. (A–F) The localization of lumenal Verm (green) and the SJ marker Cora (red) is similar in the trachea of WT (A) and *yki* mutant (C) embryos (arrow). In contrast, Verm secretion is dramatically reduced (lack of green staining) and Cora is mislocalized to the lateral membranes (arrowhead) in the SJ mutant *nrv2^23B^* (B). The SJ paracellular barrier prevents a 10 kD fluorescent dye (white) from leaking into the tracheal lumen (dashed line) in WT and *yki^B5^* trachea (D,F), but is defective in the SJ mutant *nrv2^23B^*, in which the dye fills the tracheal lumen (E). Scale bar for (A–F) in (F), 10 µm. (G–Q) Double mutant combinations of *yki^B5^* with representative mutants in known tracheal pathways all show additive effects of the mutations, suggesting that *yki* acts in a distinct pathway. (R–T) Double mutant combination of the long SJ mutant *sinu^nwu7^* and the shortest Hippo pathway mutant, *kib^4^*, reveal that *kib^4^* is epistatic to *sinu^nwu7^*, suggesting that the Hippo pathway acts downstream or in parallel to the SJ pathway. (H–Q,S–T) Maximum projections of confocal stacks. Yellow lines indicate dorsal trunk measurements. In addition to having increased length compared to either single mutant, the *yki*, *nrv2* double mutant had much more severe lumenal secretion and morphogenesis defects in the lateral tracheal branches than either single mutant (arrows vs. arrowhead in L and M). (G,R) Quantification of tracheal lengths in single and double mutant embryos. The red line in (G) marks the length of *yki^B5^* mutant trachea for comparison to double mutant combinations. Tracheal length was measured in three dimensions in confocal stacks, normalized to embryo length and then normalized to WT (*w^1118^*). Error bars are normalized s.e.m.; *: p<0.05; **: p<0.005; ***: p<0.0005. p*-*values determined using Student's *t*-test. Scale bar for (G–P) in (P), 50 µm.

Yki activity typically promotes cell division and can increase cell size, so we expected that reduced Yki activity would decrease tracheal tube length and/or diameter. Surprisingly, we found that the dorsal trunks (DTs) of stage 16 *yki* mutant embryos had a convoluted appearance and were 16% longer than DTs of wild-type (WT) embryos (Figure 1A,B,I; p<0.005). This over-elongated phenotype closely resembles the long tracheal phenotype caused by previously characterized tracheal tube-size control mutants such as *sinu* [14] and *cora* [18], [46], and *yki* trachea are indeed as long as *sinu* or *cora* mutant trachea (Fig. 1D,I; Fig. 2G, J). We confirmed that loss of *yki* activity was responsible for this over-elongated phenotype using two approaches. First, embryos homozygous for the small chromosomal deficiency Df(2R)BSC356 that completely deletes *yki* have the same long trachea phenotype as the *yki^B5^* mutant (Fig. 1I). Second, the long trachea phenotype caused by the *yki^B5^* mutation is rescued by expressing a WT form of *yki* in tracheal cells using the tracheal-specific *breathless-GAL4* (*btl-GAL4*) driver in *yki* mutant embryos (Fig. 1C,I) [47], [48]. Significantly, this result also demonstrates that *yki* is acting in tracheal cells to control tracheal tube length.

To test if Yki was not only necessary but also sufficient for controlling tube size, we expressed Yki-GFP in the trachea of WT embryos. This expression decreased tracheal length, as did overexpression of one of Yki's transcriptional co-activators, the TEAD-family member Scalloped (Sd) (Fig.1I, p<0.05 for both). Co-expression of Yki and Sd further reduced tracheal length compared to expression of Yki alone (p<0.05, Fig. 1I). Together, these results suggest that Yki has an instructive role in regulating tracheal tube length, even though Yki activity causes the opposite of what would typically be expected for a positive regulator of cell growth.

### The Hippo pathway positively regulates embryonic tracheal tube length

To determine if Yki is regulated by the Hippo pathway in the embryonic trachea, we examined zygotic mutants of the core Hippo pathway components Hpo, Warts (Wts), Salvador (Sav), Mob as tumor suppressor (Mats) and Kib [41], [49], [50]. Consistent with negative regulation of Yki by the Hippo pathway observed in other tissues, mutations in *sav*, *wts*, *mats* and *kib* had short trachea (Fig. 1F–I). Trachea in homozygous *hpo* mutants were not short, presumably due to maternal contribution (Fig. 1E,I). However, we were unable to confirm this possibility as embryos lacking both zygotic and maternal *hpo* arrested development prior to tracheal morphogenesis. Notably, the length reductions caused by mutations in Hippo pathway components were comparable to the reductions caused by overexpression of Yki and Sd, suggesting that the Hippo pathway acts through Yki to control tracheal tube size.

**Figure 3:**
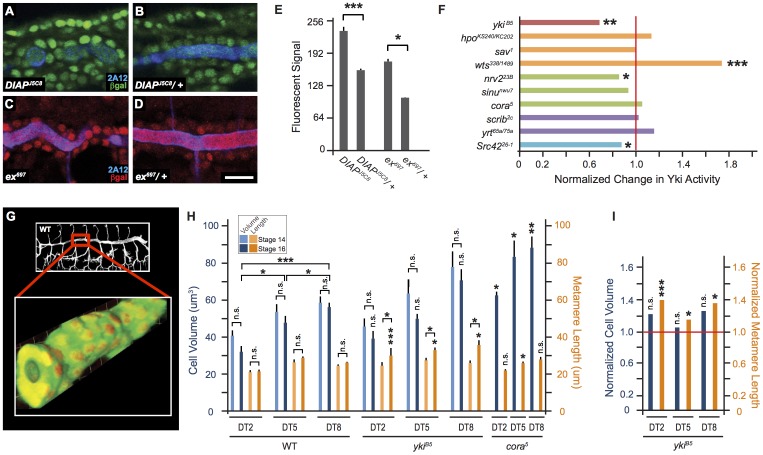
Tracheal Length Changes are Correlated with Yki Activity, but Not with Tracheal Cell Size. (A–F) Quantification of Yki reporter activity in WT and tracheal mutant backgrounds. Embryos homozygous for *DIAP-lacZ* reporter (A, green) or *ex-lacZ* (C, red) have approximately twice the signal (E) of nuclear β-gal staining in tracheal cells compared to embryos heterozygous for these reporters (B,D). (F) *yki* mutant embryos show strongly reduced reporter activity, while *wts* mutant embryos have dramatically increased reporter activity. Mutations in SJ and basolateral polarity pathways do not consistently or strongly affect reporter activity. Measurements for *yki*, *hpo*, *nrv2*, *cora*, and *Src42* used ex-lacZ, while *sav*, *wts*, *sinu*, *scrib*, and *yrt* used DIAP-lacZ. Normalized values are shown as the fold-change of the signal in trachea homozygous for a mutation compared to heterozygous control trachea on the same slide. Scale bar for (A–D) in (D), 10 µm. (G–I) Tracheal segment length is not correlated with cell volume. Cell volumes were measured in embryos expressing a cytoplasmic and nuclear GFP [48]. Embryos were fixed [8], stained for GFP to visualize tracheal cell bodies and nuclei (G, green) and for Trachealess to visualize nuclei [51]. Cell numbers, volumes, and segment lengths were analyzed using Volocity software (materials and methods). (H) Quantification of average tracheal cell volume (in light and dark blue) and metamere length (in light and dark orange) for segments 2, 5, and 8 at stages 14 (light blue and light orange) and 16 (dark blue and dark orange) for both WT and *yki^B5^*. Average cell volume and metamere length were also determined for *cora^5^* at stage 16. Notably, cell volumes consistently decrease (but are not statistically significant) between stages 14 and 16, whereas segment length increases. Statistics shown for *yki^B5^* and *cora^5^* are comparisons to WT. (I) Quantification of *yki^B5^* cell volumes and segment lengths at stage 16 normalized to WT measurements. *yki^B5^* mutant trachea are longer (orange) than WT, but cell volumes (blue) are not statistically significant different than WT. Legend in (H) also applies to (I). *, p<0.05; **, p<0.005; ***, p<0.0005; n.s., not statistically significant; p*-*values determined using Student's *t*-test. Error bars show s.e.m.

To more directly demonstrate that the Hippo pathway regulates Yki activity, we utilized transcriptional reporters that respond to Yki activity. These reporters are transposable element insertions in the Yki target genes *thread (th)* and *expanded (ex)* that produce a nuclear-localized lacZ in response to Yki activity [35]. Quantification of anti-β-gal fluorescence revealed an approximately 2-fold difference in reporter signal between trachea heterozygous or homozygous for the reporter insertions, indicating that the reporters should have sufficient dynamic range to detect changes in Yki activity. (Fig. 3A–E, Material and Methods). We verified that the *ex* reporter responds to Yki activity in the trachea by determining that reporter activity is decreased by 31% in *yki^B5^* mutant trachea (p = 0.006, Fig. 3F).

Consistent with the Hippo pathway acting through Yki to regulate tracheal length, Yki reporter activity was increased by 79% (p<0.0005) in *wts* mutant trachea (Fig. 3F), which are shorter than WT (Fig. 1A vs. G, I). Reporter activity was not significantly changed in *hpo* mutant trachea, which have WT length (Fig. 1A vs. E, I, p>0.05). However, *sav* mutant trachea, which are slightly shorter than WT trachea, did not show a significant change in reporter activity, suggesting that reporter activity as measured in stage 16 embryos may not fully reflect the spatiotemporal regulation of Yki that controls tracheal length.

### Yorkie acts separately from the SJ, basal polarity, and Src pathways

We next investigated whether *yki* mutations affect SJs or the organization of the lumenal matrix. In contrast to mutations in SJ components, such as *nrv2* [18], that cause other SJ proteins to be mislocalized around the cell periphery (e.g. Cora in Fig. 2B) and loss of paracellular barrier function (Fig. 2E), in *yki* mutants the canonical SJ protein Cora is correctly localized to the apical region of the lateral cell-cell contacts (Fig. 2C) and paracellular barrier function is intact (Fig. 2F). Further, whereas SJ mutations prevent the secretion of the lumenal matrix protein Verm (Fig. 2B), Verm secretion appears normal in *yki* mutants (Fig. 2C). Thus, the long tracheal phenotype in *yki* mutants is not caused by grossly disrupted SJs or lumenal matrix formation.

To determine if SJs could act through Yki to control trachea tube length, we tested whether Yki reporter activity was down-regulated in SJ mutants. Null mutations of the SJ components *nrv2*, *cora* and *sinu* all cause tracheal length increases similar to those caused by mutations in *yki* (Fig. 1D,I; Fig. 2G,J,L), yet reporter activity was neither consistently nor strongly altered by these SJ mutants (Fig. 3F). Consistent with Yki and SJs acting in separate pathways, double mutant combinations of the *yki^B5^* null mutation and *nrv2^23B^* null mutations have longer trachea than either the *yki^B5^* or the *nrv2^23B^* mutations alone (Fig. 2G,M) and double mutant combination of *yki^B5^* and *nrv2^23B^* had significantly more disruptions of lumenal matrix in the lateral branches (Fig. 2M, arrowhead). Interestingly, *cora^5^, yki^B5^* double mutant trachea are somewhat shorter than either *cora^5^* or *yki^B5^* alone, but still clearly longer than WT trachea (Fig. 2G,J,K). Although unexpected, this result is nonetheless consistent with SJs and Yki not acting in a single linear pathway.

To further test the relationship between the SJ and Hippo pathways, was asked if the *kib^4^* mutation, the Hippo pathway mutation that causes the shortest trachea, could suppress the long tracheal phenotype cause by the *sinu^nwu7^* mutation. *kib^4^* is completely epistatic to *sinu^nwu7^* (Fig. 2R-T), demonstrating that the Hippo pathway can profoundly influence tracheal cell shape and that the Hippo pathway acts downstream or in parallel to the SJ pathway in regulating tracheal tube size.

We also investigated whether Yki acted in either of the apicobasal polarity pathways that we had previously showed control tracheal tube length [12], [16]. Apicobasal polarity of the tracheal cells was not obviously affected in *yki* mutants, as SJ junctional components, whose localization depends on cellular polarization and adherens junction formation, were correctly localized to the apicolateral cell-cell contacts in *yki* mutants (Fig. 2C). Further, the combination of a mutation in the basolateral polarity gene *Lethal giant larvae (Lgl)* and *yki* was as long or longer than either single mutant (Fig. 2G,I,N–O). Finally, Yki reporter activity was not substantially altered in embryos lacking the basolateral polarity components *yurt* or *scrib* (Fig. 3F). Thus, it appears that *yki* acts distinctly from apicobasal polarity pathways.

We also tested if Yki acted in the pathway containing Src42, which controls both the orientation and amount of growth of tracheal cells and acts distinctly from SJs and apicobasal polarity pathways [25]. Double mutant combinations of *yki^B5^* and *Src42^26-1^*, a putative null allele, have the same short trachea phenotype of *Src42^26-1^* mutants, indicating that *Src42* is largely epistatic to *yki* (Fig. 2G,P-Q). In contrast to SJ mutations, which do not modify the *Src42* length phenotype [25], the *yki^B5^* mutation slightly lengthens the *Src42* mutant trachea (p<0.05), suggesting that Yki may act in parallel to the Src42 pathway. Consistent with this possibility, Yki reporter activity was reduced by only 10% in *Src42* mutant trachea. While it is possible that the ability of *yki* mutations to affect the *Src42* mutant phenotype results from maternal Src42 contributions, the above results suggest that Yki activity represents a distinct input into mechanisms that control tracheal tube size from the known pathways involving SJs, apicobasal polarity and Src42.

### Yorkie mutants increase tube length without increasing cell volume or number

As Yki plays a critical role in controlling cell proliferation in many tissues and can also control cell size [30], [31], [34], we investigated the possibility that loss of Yki alters tracheal cell volume or cell number. To allow comparison with normal development, we first characterized trachea cell volume in WT embryos, which had not been previously done. Tracheal cell bodies and nuclei were visualized by staining for a tracheal-GFP and for the transcription factor Trachealess (Trh) [51] (Fig. 3G and materials and methods). Cell volumes and number measurements revealed several unexpected findings. First, tracheal cell volume steadily increases along the length of the dorsal trunk. Surprisingly, at stage 16, anterior cells (DT2) have 44% less volume than posterior cells (DT8; 32 µ^3^±3 vs. 57 µ^3^±2, respectively, p<0.0005; Fig. 3H). This anterior-to-posterior differential is not observed in epidermal cells that gave rise to the tracheal cells approximately six hours earlier, but does account for anterior tracheal cells having a more squamous (flatter) profile than the more cuboidal posterior cells (epidermal cell volume for segments 2, 5 and 8, respectively: 95 µ^3^±12, 92 µ^3^±11, 103 µ^3^±8). Second, cell volume is not linearly proportional to cell length (Fig. 3H). For example, the posterior segment DT8 is only 22% longer than the anterior segment DT2, yet DT8 cells have a 60% larger volume than DT2 cells, and there are 31% more cells in DT8 than DT2 (Fig. 3H, 4J). Third, despite the length of tracheal segments increasing over the 4-hour period from stage 14–16, there is a consistent, but not statistically significant, decrease in tracheal volume over this time (Fig. 3H). Thus, while tracheal cell volume is dynamically regulated during development, tube length increases are not driven by increases in cell volume.

**Figure 4:**
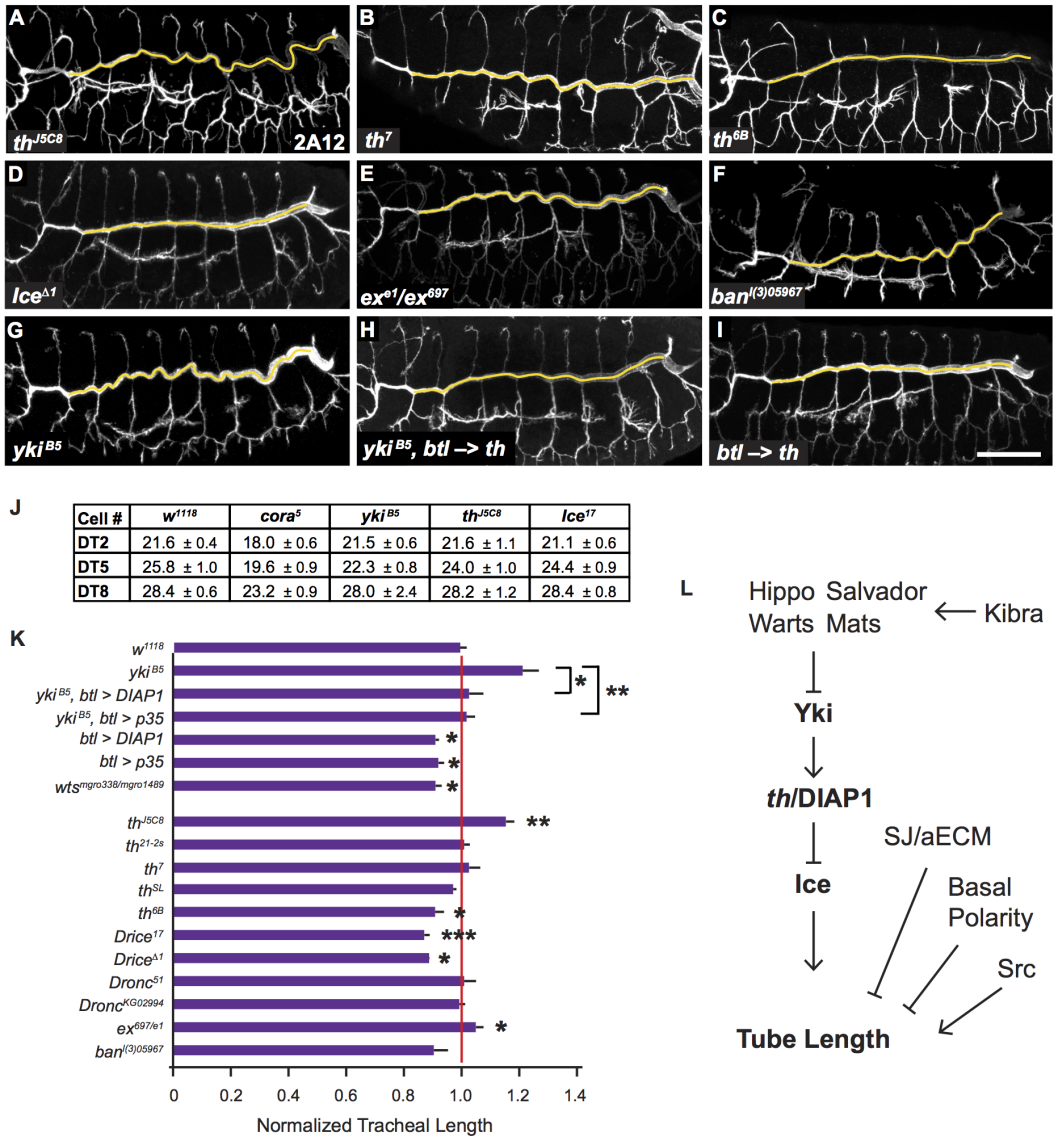
Non-apoptotic functions of *th*/DIAP1 and Ice Mediate Yki Activity in Tracheal Size Control. (A–C) *th*/DIAP1 regulates tracheal tube length. The loss-of-function allele *th^J5C8^* causes an over-elongated trachea (A) that closely resembles the *yki* phenotype (G). The hypomorphic allele *th^7^* does not alter length (B) but the gain-of-function allele *th^6B^* shortens trachea (C). (D) A loss-of-function allele of *Ice*, an effector caspase normally inhibited by DIAP1, shortens the trachea, similar to the GOF *th* allele in (C). (E–F) Mutations in other Yki targets, *ex* and *bantam*, do not cause dramatic changes in tracheal length. (G–H) Expression of *th* in the trachea of *yki^B5^* mutants is sufficient to rescue tracheal length to WT. (I) Expression of *th* in WT trachea shortens trachea, similar to the GOF *th^6B^* allele and *Ice* mutants. Yellow lines indicate dorsal trunk measurements. Lumens visualized by 2A12 staining. (J) Quantification of tracheal cell numbers in *w^1118^*, *cora^5^, yki^B5^, th^J5C8^,* and *Ice^17^* mutants. (K) Quantification of tracheal lengths for *th*, *Ice*, *ex* and *ban* mutants, and *th* and p35 expression experiments. Tracheal length was measured in three dimensions in confocal stacks, normalized to embryo length and then normalized to WT (*w^1118^*, red line). Error bars show normalized s.e.m.; *: p<0.05; **: p<0.005; ***: p<0.0005. p*-*values determined using Student's *t*-test. Scale bar for (A–H) in (H), 50 µm. (L) Model of genetic pathways regulating tracheal tube length.

Having established a baseline of WT development, we determined tracheal cell number and volume in *yki* mutants and, for comparison, the SJ mutant *cor*. Consistent with the almost complete absence of cell death or division during most of tracheal morphogenesis [9], [10], cell number was not significantly altered in *yki* mutants (Fig. 4J). Tracheal cell volume in *yki* mutants was also not statistically different than in WT (Fig. 3I). Thus, the increased tracheal length in *yki* mutants is not driven by increased tracheal cell volume. Surprisingly, in the SJ mutant *cor*, cell volumes were significantly increased compared to WT (Fig. 3H; p<0.05 for DT2 and DT5, p<0.005 for DT8), providing further evidence that Yki does not act in the same pathway as SJ components. In combination, these results suggest that while cell volume is dynamically regulated in the tracheal system, cell volume changes do not drive tube length increases.

### Yki acts through *thread*/DIAP to control tracheal tube length

Since Yki does not appear to control tracheal size by regulating cell growth, we examined embryos mutant for the known Yki transcriptional targets: *thread* (*th*, which encodes the protein DIAP1- Drosophila Inhibitor of Apoptosis), *expanded* (*ex*), and *bantam* (*ban*). Tracheal length was not substantially changed in *ban* or *ex* mutants (Fig. 4E,F,K). However, embryos homozygous for the reduction-of-function *th^J5C8^* mutation, which results from a transposable element insertion in the *th* 5′ UTR [52], [53], had dramatically longer trachea, comparable to length increases in *yki^B5^* mutants (p<0.005; Fig. 4A,K). We attempted to confirm this result using additional loss-of-function alleles of *th*: *th^4^*, which substitutes a tyrosine for an invariant histidine in the BIR2 domain, and *th^7^*, which is a small deletion the removes part of the BIR2 domain [54]. Unfortunately, most homozygous *th^4^* embryos had severe morphogenesis defects and it was not possible to meaningfully determine tracheal length of this genotype (data not shown). On the other hand, *th^7^* mutants had normal embryonic morphology, but also had WT tracheal length, presumably because of a combination of maternal contribution and *th^7^* being a partial loss-of-function mutation.

To further investigate the role of DIAP in tracheal size control, we also examined embryos homozygous for three gain-of-function mutations in the *th* locus, each of which has a different spectrum of interactions with the pro-apoptotic proteins Grim, Reaper and Hid, and with the caspases Dronc and Ice [54]–[56]. We examined *th^SL^*, which is a valine to methionine substitution in the BIR1 domain [54], *th^21-2s^* which is a semi-lethal allele resulting from a proline to serine change also in the BIR1 domain [55] and *th^6B^* which substitutes tyrosine for an invariant cysteine in the RING domain [54]. Tracheal length was normal in homozygous *th^SL^* and *th^21-2s^* st. 16 embryos. However embryos homozygous for *th^6B^* had shortened trachea that were as short as the strongest SWH mutants (Fig. 4C,I; [57]). Since the *th^6B^* can act as a dominant negative mutation with regard to Hid induced-killing in the eye [54], this result supports the model that DIAP mediates the effects of Yki activity and implicates Hid as a potential regulator of tracheal length. Notably, *th^SL^* mutation does not act as dominant negative with regard to Hid [54]. The significance of the semi-viable *th^21-2s^* mutation not causing a tracheal phenotype is unclear since the mutation only causes partial lethality and was scored by Goyal *et al.* as a weak suppressor of Hid in the eye. Although the genetics of DIAP1 mutations is complex [56], and further complicated by maternal contribution in the embryo, these results clearly implicate DIAP as a downstream effector of the Yki in tracheal size control.

To more directly assess the role of DIAP in tracheal size control, we expressed WT *th* in the trachea of otherwise WT embryos. Consistent with *th* mediating the effects of *yki* on tracheal size, *th* expression resulted in shortened tracheal tubes that were approximately the same length as Hippo pathway mutants (Fig. 4I,K). To confirm the overall relationship of the Hippo pathway and *th*, we examined *kib^4^, th^J5C8^* double mutants (a short *kib* mutant in combination with a long *th* mutant), however the mid-body tracheal segments were grossly defective in these embryos and we were unable to determine tracheal length (data not shown). Critically, expressing *th* in the trachea of *yki^B5^* mutants restored tracheal length to WT (Fig. 4H,K), strongly supporting the model that *th*/DIAP1 is the primary mediator of Yki function in tube size control.

### A non-apoptotic function of the caspase Ice mediates tracheal tube size control

How does the *th*/DIAP1 control tube size? A critical function of DIAP1 is to regulate cell death by binding and inhibiting the pro-apoptotic caspases Dronc and Ice [58], [59]. We tested if caspases control tracheal size by characterizing the phenotypes of mutations in *Dronc* and *Ice.* Tracheal length was not altered by the *Dronc^51^* null mutation, however, the loss-of-function *Ice^17^* and *Ice^Δ1^* mutations decreased tracheal length as much as the *th^6B^* and *wts^338/1489^* mutations (Fig. 4C–D,K). Given that Dronc is thought to be required to activate Ice (reviewed in [56]), it is somewhat surprising that Ice but not Dronc mutations affect tracheal length. However, it is likely that a role for Dronc in tracheal size control is obscured by the significant maternal contribution of Dronc [56]. To confirm a role for Ice and its proteolytic activity in size control, we used the tracheal specific driver *btl*-Gal4 to express baculovirus p35, a well-characterized caspase inhibitor that covalently attaches to and inhibits Ice. p35 expression decreased tracheal length similar to *Ice* and *th^6B^* mutations. Because p35 blocks proteolytic activity of Ice without necessarily eliminating Ice protein, this result suggests the proteolytic activity of Ice is required for tracheal size control, even though Baer *et al.* did not observe significant staining of an antiserum that binds cleaved caspases in the dorsal trunks of late stage embryos [10]. Strikingly, expressing p35 in *yki^B5^* mutant trachea restored tracheal length to WT, strongly supporting the model that Yki acts through DIAP1 and subsequently Ice to regulate tube size (Fig. 4K).

To confirm that changes in overall tracheal cell number were not influencing tracheal system length, we counted the tracheal cells in *yki*, *th* and *Ice* mutants (Fig. 4J). Tracheal cell numbers in these mutants were within the WT range and not correlated with tracheal length (Fig. 4J and [8], [9]). Thus, control of epithelial tube length by Yki-DIAP1-Ice is a non-apoptotic function of this evolutionarily conserved cell death pathway.

## Discussion

The Hippo-Yki/YAP/TAZ pathway has been well established as the regulator of organ size via cell proliferation and cell death. However, this pathway also has broader roles, including regulating cell-cycle exit and differentiation, acting as a switch in cell fate decisions, and controlling signaling pathways [60], [61]. To date there has been limited investigation of non-proliferative/apoptotic roles of the Hippo pathway or Yki/TAZ/YAP in tubulogenesis and cell shape control, but interesting and unexpected findings are emerging. Mice and zebrafish lacking TAZ develop cystic kidneys due to an abnormal accumulation of polycystin-2, which TAZ normally regulates through an ubiquitin ligase complex [62]. Tissue specific inactivation of YAP in developing mouse nephrons severely disrupts nephron morphogenesis, without affecting either cell proliferation or cell death [63]. While the altered expression of genes that mediate tubule morphogenesis in YAP-deficient cells likely contributes to abnormal nephron morphogenesis, failure to activate non-apoptotic functions of cell death genes may also contribute. The Drosophila tracheal system provides a powerful model system for investigating non-proliferative/apoptotic roles of the Hippo pathway and Yki/YAP/TAZ in morphogenesis.

One puzzling aspect of our results is that previous work by Ghabrial *et al*. showed that single cell clones of a Wts mutation in the larval dorsal trunk caused what they described as a “general overgrowth” phenotype, and what appears to be a roughly isotropic increase in tracheal cell apical surface [64]. This increase is in marked contrast to the reduction in apical surface area we observed in the shortened dorsal trunks of homozygous *wts^338/1489^* embryos. Explanations for this discrepancy fall into two general categories. First, it may be the case that the Hippo pathway acts on targets other than, or in addition to, Yki, that differ between embryos and larvae. Similarly, Hippo may regulate Yki equivalently in larvae and embryos, but Yki may have different targets in the two stages. Second, and perhaps more interestingly, the Hippo pathway may mediate cell-cell communication that regulates apical membrane growth, and a single cell lacking Hippo pathway activity behaves differently depending on whether its neighbors have a functional Hippo pathway. In this model, similar results would be obtained in embryos and larvae as long as equivalent experiments are performed (single cell clones versus entire tissue). Resolving this discrepancy will require making single cell clones of Hippo pathway components in embryonic trachea, and reducing Hippo pathway activity in all tracheal cells during larval development.

While additional details of the roles of Hippo and Yki remain to be elucidated, our results clearly demonstrate a non-apoptotic function of DIAP1 and Ice in embryonic epithelial tube size control. What is this non-apoptotic function? An attractive possibility is that it would be related to the DIAP1-dependent, but non-apoptotic, process that regulates migration of border cell migration during oogenesis. However, in contrast to tracheal size control, border cell migration does not require Ice, and is not affected by expression of p35, which binds to and inhibits Ice but not Dronc [65]. Moreover, Lucas *et al.* recently showed that the Hippo pathway acts through the actin regulatory protein Ena to activate F-actin Capping protein activity on inner membranes and restrict F-actin polymerization [66]. Since our results show that tracheal size control depends on DIAP1 and Ice, these results argue that the Hippo pathway and DIAP have distinct roles in border cell migration and tracheal size control.

A possible function of DIAP1 and Ice to regulate tracheal membrane involves SREBP/HLH106, the sterol response element binding protein that is a transcription factor required for production of membrane lipid. Interestingly, SREBP is translated as a plasma membrane-bound form that is cleaved by Ice to liberate the functional transcription factor [67]. In the tracheal system, non-apoptotic Ice activity may activate SREBP to promote membrane expansion. Another intriguing possibility is that tracheal growth could be related to non-apoptotic functions of caspases-3 that enable melanoma cells to generate tubes via “vascular mimicry”, the process by which non-endothelial tumor cells form tubes that connect the tumor to the vascular system to facilitate tumor growth. However, the role of caspase-3 in mammalian tube formation pathway remains to be determined [68], [69]. Plausible pathways that could be regulated by non-apoptotic functions of caspases include apicobasal polarity, which we have previously showed control cell length [12], [16], and the endomembrane system, which could potentially mediate changes in apical surface area, cell shape and tube size.

Further work will be required to delineate the targets and logic of caspase-mediated tube-size control in the trachea, but the genetic tractability of the Drosophila trachea system make it an excellent system for investigating the non-canonical functions of Yki, DIAP1 and Ice in cell shape control and organ morphogenesis.

## Materials and Methods

### Fly reagents

Fly stocks and alleles were obtained from Bloomington *Drosophila* Stock Center [70] or were generously provided by A. Bergmann (*Ice^17^*), R. Carthew *(yw hsFLP; FRT42D, FRT42B ovo^D1^/MS(2)M1, Sp/CyO*), R. Fehon (*yki^B5^;UAS-FLAG-yki*), A. Ghabriel (*wts^miracle-gro338^*, *wts^miracle-gro1483^*, *wts^miracle-gro1489^*), G. Halder (*yki^B5^*, *ex^e1^*), I. Hariharan *(wts^MGH1^*, *sav^1^*), B. Hay (*Ice^Δ1^*), R. Holmgren (*UAS-p35*), J. Kennison (*th^7^*), K. Moberg (*th^J5C8^*, *ex^697^*), H. Steller (*th^21-2s^*), H. Stocker (*kib^4^*), and Kristin White (*th^6B^, th^SL^*). Fly stocks were maintained at room temperature and were balanced with *dfd*-GMR-YFP marked balancer chromosomes [71], which also allowed genotyping of embryos. Imaging performed at stage 16 unless otherwise noted. Germline mosaic clones were attempted using *the hpo^KS240^* allele and published approaches [72]–[74].

### Immunohistochemistry

The following antibodies were used: mouse (ms) anti-2A12 1∶2, rabbit (rb) anti-Verm 1∶300 (S. Luschnig), rat anti-Trachealess 1∶50 (B. Shilo [51]), guinea pig (gp) anti-Cora 1∶10000 (R. Fehon), rb anti-β-galactosidase 1∶75 (Capel), ms anti-GFP (Molecular Probes, A11120), and rb anti-GFP (Molecular Probes, A11122). Alexa Fluor 488, 546, and 647 were used for secondary antibodies (Molecular Probes). The lumenal antibody 2A12, embryo fixation, staining, and staging procedures were performed as previously described [8]. All embryos were imaged with a Leica SP2 or SP5 confocal microscope. Control heterozygous embryos were imaged on the same slide as the experimental homozygous embryos using the same laser settings such that relative signal levels could be directly compared. For Yki reporter activity experiments, lasers were set such that in a control embryo, the maximum signal for pixels in tracheal nuclei did not saturate the detector.

### Measurements

Confocal image analysis was performed using the Perkin Elmer Volocity software program. Normalized tracheal lengths were calculated by measuring the length of the dorsal trunk from DB2 to DB10 and dividing by embryo body length. For Yki activity experiments, signal levels of 20–30 nuclei in DT7 and DT8 tracheal cells (3–5 sections/segment) per embryo were measured and averaged. Tracheal metamere volumes were determined from confocal sections of fixed embryos that had expressed a cytoplasmic and nuclear GFP in the trachea. GFP was visualized with a fluorescent secondary antibody. Trachea were automatically detected as “objects” with a fluorescence intensity above a threshold value. To fill missing volumes in the cell bodies due to non-uniform expression of GFP, automatic functions of the Volocity software were used to “dilate”, “close” and “fill” spaces within the confocal stack. This resulted in a contiguous filled object for the cell bodies of the trachea. A representative image of processed trachea is shown in Fig. 3G. Tracheal metameres were manually segmented and the volume of segmented object was calculated. For statistical analyses, p-values were calculated using Student's *t*-test with two-tailed unpaired variance.

### Dye exclusion assay

Paracellular barrier function of tracheal SJs was assayed as previously described by injecting a 10 kD Texas Red-conjugated dextran (Molecular Probes) into the body cavities of stage 16 embryos [75]. Injected embryos were imaged on a Leica TCS SP2 confocal microscope within 20 min of injection.
